# PTP4A2 Promotes Glioblastoma Progression and Macrophage Polarization under Microenvironmental Pressure

**DOI:** 10.1158/2767-9764.CRC-23-0334

**Published:** 2024-07-11

**Authors:** Tiffanie Chouleur, Andrea Emanuelli, Wilfried Souleyreau, Marie-Alix Derieppe, Téo Leboucq, Serge Hardy, Thomas Mathivet, Michel L. Tremblay, Andreas Bikfalvi

**Affiliations:** 1 INSERM U1312 BRIC, Université de Bordeaux, Pessac, France.; 2 Rosalind and Morris Goodman Cancer Institute, McGill University, Montreal, Canada.; 3 Animalerie Mutualisée, Service Commun des Animaleries, Université de Bordeaux Bordeaux, France.

## Abstract

Phosphatase of regenerating liver 2 (also known as *PTP4A2*) has been linked to cancer progression. Still, its exact role in glioblastoma (GBM), the most aggressive type of primary brain tumor, remains elusive. In this study, we report that pharmacologic treatment using JMS-053, a pan–phosphatase of regenerating liver inhibitor, inhibits GBM cell viability and spheroid growth. We also show that *PTP4A2* is associated with a poor prognosis in gliomas, and its expression correlates with GBM aggressiveness. Using a GBM orthotopic xenograft model, we show that *PTP4A2* overexpression promotes tumor growth and reduces mouse survival. Furthermore, *PTP4A2* deletion leads to increased apoptosis and proinflammatory signals. Using a syngeneic GBM model, we show that depletion of *PTP4A2* reduces tumor growth and induces a shift in the tumor microenvironment (TME) toward an immunosuppressive state. *In vitro* assays show that cell proliferation is not affected in *PTP4A2*-deficient or -overexpressing cells, highlighting the importance of the microenvironment in *PTP4A2* functions. Collectively, our results indicate that *PTP4A2* promotes GBM growth in response to microenvironmental pressure and support the rationale for targeting *PTP4A2* as a therapeutic strategy against GBM.

**Significance:**

High levels of *PTP4A2* are associated with poor outcomes in patients with glioma and in mouse models. *PTP4A2* depletion increases apoptosis and proinflammatory signals in GBM xenograft models, significantly impacts tumor growth, and rewires the TME in an immunocompetent host. *PTP4A2* effects in GBM are dependent on the presence of the TME.

## Introduction

Glioblastoma (GBM) is the most common and clinically aggressive primary brain tumor (1). This tumor is characterized by highly proliferative cells surrounding a necrotic core, rich vascularization, and tumor cell invasion. The treatment established by Stupp and colleagues (2) in 2005 consists of maximal surgical resection of the tumor followed by concomitant radiotherapy and chemotherapy and is still the standard of care. No additional survival benefit has been brought by novel therapies since, resulting in a dismal median survival of approximately 15 months (3). Most importantly, GBM forms a heterogeneous group of cancers that differs between individuals but also displays intratumor heterogeneity (4, 5). The multilevel heterogeneity of GBM is most likely responsible for therapeutic resistance, leading to the inevitable tumor recurrence. In this context, it seems essential to develop new therapeutic strategies against GBM. A potential means for this purpose is to identify new targets that could be used in monotherapy as well as in combination with existing therapies.

The phosphatase of regenerating liver 2 (PRL2), encoded by *PTP4A2*, is a protein tyrosine phosphatase that belongs to the dual specificity phosphatase subfamily (6). As such, it dephosphorylates both tyrosine and/or serine/threonine residues within the substrate. Although the *in vitro* activity of PRL2 is low (7), substrates have been identified for this protein tyrosine phosphatase (8, 9). In addition, *PTP4A2* has been associated with the control of magnesium homeostasis through an interaction with the cyclin M (CNNM) magnesium transport regulator family in a substrate-independent manner. The PRL phosphatase family is known to be overexpressed or to promote oncogenesis in multiple cancer types including lung, breast, kidney, and colorectal cancers, melanomas, and gliomas (10–12). However, the role of *PTP4A2* in GBM progression has not been thoroughly investigated.

Here, we provide evidence that pharmacologic inhibition of the PRLs could constitute a strategy to target GBM. We show that *PTP4A2* is upregulated in GBM and is associated with aggressiveness and poor patient survival. In a xenograft model, *PTP4A2* modulates GBM growth and apoptosis. Finally, the decrease in *PTP4A2* levels in GBM cells affects the tumor microenvironment (TME) by increasing proinflammatory signals.

## Materials and Methods

### Clinical databases

The Cancer Genome Atlas (TCGA) and Genotype-Tissue Expression (GTEx) data were downloaded via the Xena Browser (https://xenabrowser.net/). For survival analysis, TCGA Low-Grade Glioma (LGG) and GBM data of overall survival and gene expression were used to perform survival analysis in RStudio (RRID: SCR_001905) with the packages survminer (RRID: SCR_021094), survival (RRID: SCR_021137), and ggplot2 (RRID: SCR_014601). Data were split into two groups based on high and low gene expression levels with an optimal cut-point. *P* values correspond to log-rank *P* values. TCGA GBM and LGG and GTEx brain gene expression data were used to perform comparative analysis of gene expression in tumor versus normal tissue. Analysis was performed in R studio with the packages RVAideMemoire (RRID: SCR_015657), FSA (RRID: SCR_016114), pheatmap (RRID: SCR_016418), and ggplot2. Single cell RNA sequence (scRNA-seq) data from GBM specimens were analyzed via https://singlecell.broadinstitute.org/ (13) to generate a cellular state hierarchy plot showing *PTP4A2* expression.

### Patient-derived GBM cells

P3, NCH644, NCH601, and NCH421k cells were kindly provided by Prof. Rolf Bjerkvig (University of Bergen, Bergen, Norway) and cultured as suspension cells in Neurobasal medium (NBM, Gibco) supplemented with B-27 (Gibco), 10 ng/µL FGF-2 (Proteintech), and 5,000 U/µL heparin (Sigma). 83-Mes, 157-PN, and 1123-Mes were kindly provided by Dr. Janusz Rak (McGill University, Montreal, Canada) and cultured as suspension cells in DMEM/F12 (Gibco) supplemented with B-27 (Gibco), GlutaMAX (Gibco), 20 ng/µL FGF-2 (Proteintech), 20 ng/µL EGF (Proteintech), and 10,000 U/µL heparin (Sigma). All the cellular models used in this study were cultured as neurospheres and were previously characterized (14, 15). *PTP4A2*-knockout (*PTP4A2-*KO) and *PTP4A2*-overexpressing (*PTP4A2*-OE) P3 cells were obtained by infection of cells with lentivirus particles carrying the lenti-CRISPRv2 or pLenti6 plasmid, respectively, without clonal selection. As for 1123-Mes and 157-PN cells, clonal selection was performed, and the cells used for the experiments were a pool of *PTP4A2*-KO clones.

### Mouse cell line

CT2A cells were kindly provided by Dr. Thomas N. Seyfried (Biology Department, Boston College, USA; refs. 16, 17) supplemented with 10% FBS (Life Technologies), 1% penicillin/streptomycin (Life Technologies), and 1% glutamine (Life Technologies).

### *Mycoplasma* testing and handling of cell lines

Cell lines were tested for *Mycoplasma* using genomic PCR (probes: Fwd-GGCGAATGGGTGAGTAACAC; Rev-CGGATAACGCTTGCGACCTAT) on a monthly basis and prior to any cryopreservation process. Cryopreserved cells were obtained and frozen at passage 5 and were maintained in culture until passages 15 to 20.

### Proliferation assays

For viability assay, CellTiter-Glo 3D cell viability assay (Promega) was used according to the manufacturer’s instructions. For 3D spheroid growth rate assay, P3 cells were seeded at 10^5^ cells in 200 μL of complete NBM containing 0.4% methylcellulose in a 96-well round-bottom plate. Spheroid growth was followed for 2 weeks by taking pictures using a videomicroscope, and areas were measured using Fiji (18). For 3D spheroid growth rate assay in 1123-Mes and 157-PN cells, 1,000 cells were seeded in 200 μL of complete DMEM/F12 containing 0.4% methylcellulose in a 96-well round-bottom plate. Spheroid growth was followed for 1 week in an automated imaging system (Incucyte) and analyzed using Incucyte S3 2019A software.

### Migration assay

P3 cells were seeded on Matrigel coating (Corning, CLS356252) at confluency in an Imagelock 96-well plate (Sartorius). Cells were starved overnight in NBM supplemented with B27 only. A scratch was created using a WoundMaker (Sartorius). Cells were washed with PBS to remove debris and incubated in complete NBM in Incucyte in which a picture was taken every 2 hours for 48 hours. The migration rate is expressed as wound closure over time calculated using the formula wound closure= [wound(T0)-wound(t)]×100/wound(T0).

### Spheroid invasion assay

Spheroids of 10^5^ (for P3) or 1,000 (for 1123-Mes and 157-PN) cells were included into 100 µL of collagen type I gel at 1 mg/mL in a 96-well plate. After 40 minutes of polymerization at 37°C, 100 µL of medium was added on the gel. Images were taken using a videomicroscope after 24 hours of invasion and quantified using Fiji. Invasion was calculated as follows:

invasion rate=[invasive area(T24h)/core area(T24h)] and expressed as *z* scores.

### PRL inhibitor

The PRL inhibitor JMS-053 and its control counterpart JMS-038 were kindly provided by Dr. John S. Lazo. For cell viability assay, cells were seeded in 96-well plates in complete medium supplemented with 0.1 to 40 µmol/L of JMS-053 or JMS-038 in DMSO. The plates were incubated for 5 days, and an ATP luminescent cell viability assay was used according to the manufacturer’s instructions (CellTiter-Glo 3D cell viability assay, Promega). Alternatively, spheroids of 1,000 cells were incubated with the compounds using the same concentrations as above, for 5 to 7 days, depending on the cells used as shown in the figures. Spheroid 3D growth was recorded as described above.

### qPCR

Total RNAs were extracted using an acid guanidinium thiocyanate–phenol–chloroform extraction method (TRI Reagent MRC, TR 118) and reverse-transcribed into cDNA using the High-Capacity cDNA Reverse Transcription Kit (Applied Biosystems). qPCR reactions were performed using EurobioGreen (Eurobio, GAEMMX02H), according to the manufacturer’s instructions. The relative expression of the genes was calculated using the 2^−ΔΔCT^ method and with reference genes indicated in the figure legends. Primer sequences are listed in Supplementary Table S1.

### *In vivo* experiments

Male Rag2^−/−^ γc^−/−^ mice and C57Black6 mice were housed and treated in the animal facility at Bordeaux University. All animal procedures were done according to the institutional guidelines and approved by the local Ethics Committee (ref 2019031909389750). For xenograft experiments, five spheroids of 10,000 P3 cells or 1,000 1123-Mes cells were orthotopically implanted in randomly chosen mice (8–12 weeks old). For syngeneic implantations, 50,000 CT2A cells were implanted in randomly chosen mice (8–12 weeks old). Cells or spheroids were stereotactically implanted into the right cerebral cortex (2.2 mm left from bregma, 3 mm deep) using a Hamilton syringe fitted with a needle. For survival experiments, each mouse was sacrificed at an endpoint (neurologic signs or weight loss) to compare tumors at maximal development. For the other experiments, mice were sacrificed at the same time to compare tumor development. For each experiment, brains were collected for protein extraction, RNA extraction, and histology. Each experiment was carried out with a minimum of 5 to 8 mice per group.

### Immunoblotting

Cell or tissue lysates were extracted in RIPA buffer (50 mmol/L Tris-HCl (pH = 7.4), 150 mmol/L NaCl, 0.5% deoxycholic acid, 1% Triton X-100, 0.1% SDS, protease inhibitor, and PhosphoPLUS) or tissue extraction buffer (50 mmol/L Tris-HCl (pH = 7.4), 140 mmol/L KCl, 1 mmol/L Ethylene Glycol-bis-(beta-aminoethylether)-N,N,N′,N′-tetraacetic Acid (EDTA), 1 mmol/L EGTA, 1% Triton X-100, protease inhibitor, PhosphoPLUS, and 1 mmol/L phenylmethylsulfonylfluoride). Protein quantification was conducted via bicinchoninic acid (BCA) protein assay (Pierce, 23225). Proteins were separated in SDS-PAGE and transferred onto a polyvinylidene difluoride membrane (Amersham). The membranes were blocked in a blocking buffer containing 5% BSA and 0.1% Tween-20 for 1 hour and incubated with a primary antibody overnight at 4°C. The membranes were washed and incubated for 1 hour with secondary antibodies (LI-COR) and imaged using an infrared fluorescent scanner (Odyssey, LI-COR Biosciences).

### Histology

Mouse brains were processed for formalin fixation and paraffin embedding. In detail, brains were fixed in formalin 4% for 24 to 48 hours at 4°C progressively dehydrated in ethanol, incubated in toluene, and embedded in paraffin. Sections of 5 μm were used for immunostaining. For immunostaining, sections were deparaffinized in toluene and rehydrated gradually in ethanol. Sections were incubated with antigen retrieval buffer (10 mmol/L citrate buffer pH 6), heated in a microwave for 10 minutes, cooled down to room temperature (RT), and then rinsed in dH_2_O. Slides were saturated for 1 hour in 3% BSA in PBS. Primary antibodies (See Supplementary Table S1) were dissolved in 1% BSA in PBS and incubated overnight at 4°C. After washes, slices were incubated with secondary antibodies for 1 hour at RT. 4′,6-Diamidino-2-phenylindole (DAPI) was diluted in PBS and incubated for 10 minutes. Slices were mounted in an antifading medium (ProLong, Thermo Fisher Scientific) and dried at RT before imaging.

Alternatively, flash-frozen mouse brains were cut into 10-µm sections using a cryostat. Sections were then used for immunostaining. For immunostaining, sections were fixed in 4% paraformaldehyde for 10 minutes and saturated for 1 hour in 3% BSA in PBS. The sections were incubated with primary and secondary antibodies as described for formalin-fixed, paraffin-embedded sections.

To label dead tumor cells, terminal deoxynucleotidyl transferase–mediated dUTP nick end labeling (TUNEL) fluorescence staining was performed on frozen sections and formalin-fixed, paraffin-embedded sections following the manufacturer’s protocol (Abcam, ab66110).

Images were acquired using a slide scanner (Hamamatsu NanoZoomer 2.0HT) at 20× or using a upright microscope (DM4 B, Leica) equipped with a camera (DFC7000 T, Leica), and 3 to 6 fields per tumor of 5 to 8 mice per condition were analyzed in Fiji.

### Statistical analysis

All data represented herein were performed in biological replicates of three or more and are presented as the mean ± SEM, unless otherwise indicated, and analyzed using RStudio version 4.2 (https://www.r-project.org/). For each figure, statistical tests were justified as appropriate (homogeneity of distributions and variances were assessed), and the following *P* values were displayed: *, *P* < 0.05; **, *P* < 0.01; ***, *P* < 0.001; ****, *P* < 0.0001, ns > 0.05. In detail, the Kruskal–Wallis test followed by the Dunn test was used to assess statistically significant differences between three or more groups, whereas the permutation *t* test and Mann–Whitney U test were used to compare two groups. Immunostaining and Western blot figures show a representative experiment. However, all quantitative graphs show the results from the pooling of a minimum of five biologically independent samples. Statistical analyses of all sample groups were performed and corrected by Welch and Bonferroni *post hoc* tests if appropriate.

### Data availability

TCGA and GTEx data can be downloaded via the Xena Browser (https://xenabrowser.net/). All other data may be obtained upon request to A. Bikfalvi (andreas.bikfalvi@u-bordeaux.fr).

## Results

### Pharmacologic inhibition of all three PRLs inhibits GBM cell viability and spheroid growth

**Figure 1:**
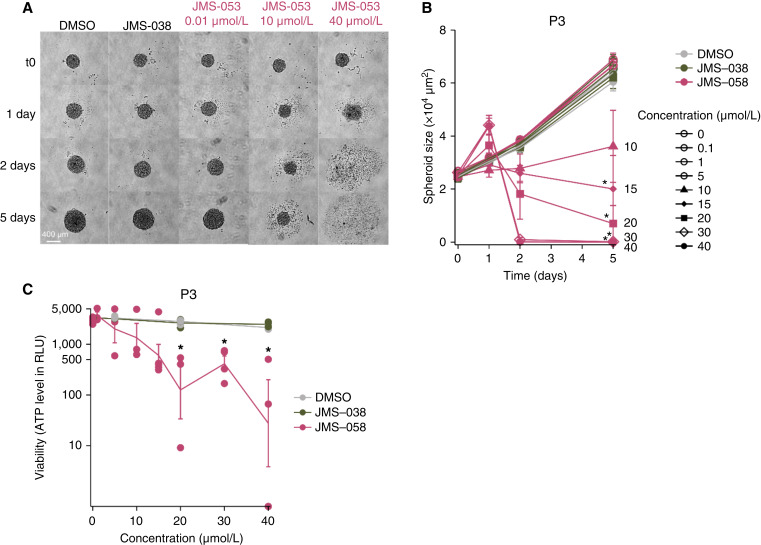
Inhibition of the three PRLs by JMS-053 in P3 spheroids. **A,** Representative pictures of spheroid growth over 5 days of P3 cells treated with the PRL inhibitor JMS-053 (pink), the control compound JMS-038 (green), or the vehicle DMSO (grey). **B,** Quantification of P3 spheroid growth. *n* = 3. Kruskal–Wallis test followed by Dunn tests. **C,** P3 spheroid viability after 5 days of treatment with JMS-053 (pink), JMS-038 (green), or DMSO (grey) as shown by ATP level. IC_50_, around 15 µmol/L. *n* = 3. Kruskal–Wallis test followed by Dunn tests.

Given the growing evidence of PRL involvement in various cancer types, we first assessed if they could be potential targets for drug therapy in GBM. As the PRLs potentially compensate for the functions of each other, we initially hypothesized that targeting this entire subfamily would be more relevant for cancer therapy. We thus decided to use the chemical inhibitor JMS-053, which inhibits all three PRLs (PRL1, PRL2, and PRL3) with a reported IC_50_ ranging from 1 to 25 µmol/L in *in vitro* assays on ovarian cancer cell lines (19). Three different human GBM cell lines were treated with various concentrations of JMS-053 or the control compound JMS-038 or carrier control DMSO for 48 hours. The inactive control congener JMS-038 is a close structural analogue of JMS-053 that is inactive against all three PRL family members at concentrations as high as 100 µmol/L (19, 20). The inhibition of PRLs reduced P3 cell viability with an IC_50_ of around 10 µmol/L (Supplementary Fig. S1). The inhibitory effect was also observed on P3 spheroid growth monitored for 5 days as well as viability quantified by the ATP level (Fig. 1A–C). The negative control compound JMS-038 did not inhibit the growth and viability at concentrations as high as 40 µmol/L. Similarly, PRL inhibition reduced spheroid growth in the 157-PN and 1123-Mes GBM models at concentrations of JMS-053 above 5 µmol/L (Supplementary Fig. S2A and S2B). This initial pharmacologic assessment suggests a potential therapeutic interest in targeting the PRLs in GBM.

### Upregulation of *PTP4A2* gene expression in gliomas is associated with aggressiveness and poor prognosis

**Figure 2:**
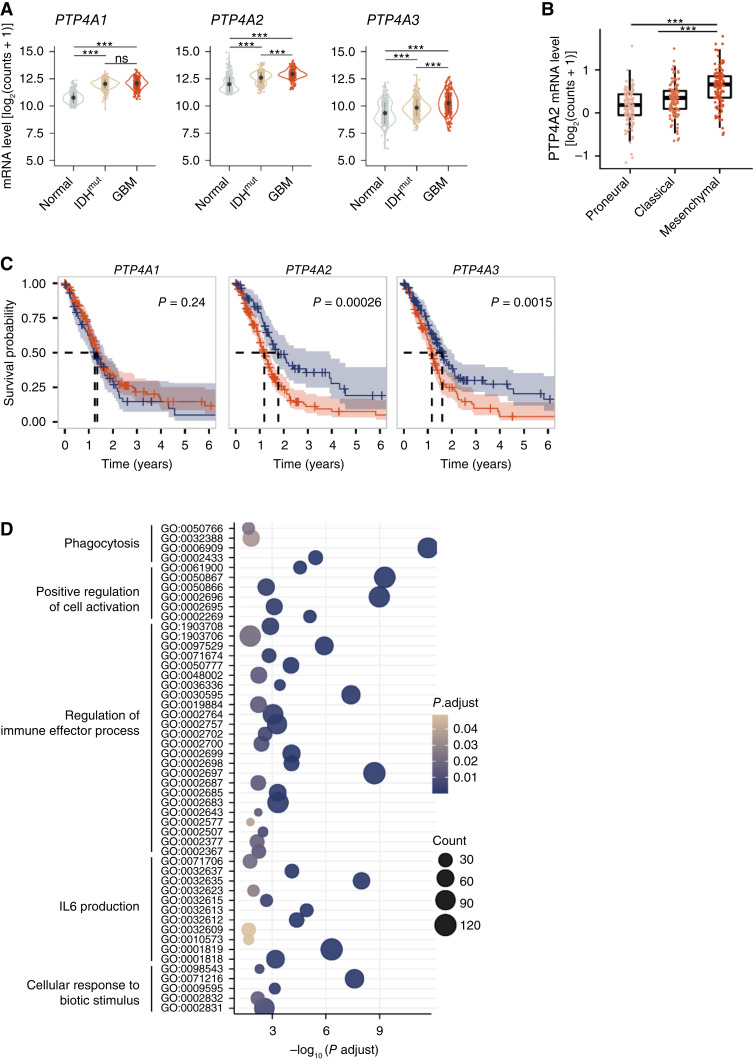
High levels of *PTP4A2* in gliomas are associated with tumor aggressiveness and poor overall survival of patients. **A,***PTP4A1*, *2*, and *3* mRNA levels of normal samples (nontumor from the GTEx database), IDH-mutant (IDH^mut^) gliomas, and IDH wild-type GBM (from TCGA). **B,***PTP4A2* mRNA levels in GBM subtypes: proneural, classical, and mesenchymal, the most aggressive. **C,** Kaplan–Meier curve of overall survival of patients with GBM stratified according to the expression level of *PTP4A1*, *2*, and *3* (from TCGA). Log-rank *P* value = 0.001. *PTP4A1* high, *n* = 179; *PTP4A1* low, *n* = 76; *PTP4A2* high, *n* = 173; *PTP4A2* low, *n* = 82; *PTP4A3* high, *n* = 124; *PTP4A3* low, *n* = 132. **D,** Enrichment in biological pathways involving *PTP4A2-*correlated genes in TCGA GBM data. Only the first five clusters of GO terms are displayed.

To identify the specific PRL that might play a more important role in GBM development, we examined the expression level of *PTP4As* in gliomas and their correlation with patient survival using TCGA and GTEx data. We found that the *PTP4As* are upregulated in both isocitrate dehydrogenase (IDH)–mutant gliomas and GBM (IDH wild-type) compared with normal brain tissue with *PTP4A2* being the most expressed (Fig. 2A). *PTP4A2* is more specifically correlated with the Verhaak mesenchymal subtype of GBM (Fig. 2B; ref. 21). PTP4A2 expression is homogeneous among cellular states as defined by Neftel and colleagues (13) with a slight decrease in the oligodendrocytic precursor cell–like cellular state (Supplementary Fig. S3A). *PTP4A2* was also upregulated in some GBM cell lines including P3 cells, 1123-Mes, and 157-PN compared with normal human astrocytes (NHA-TS; Supplementary Fig. S3B). Moreover, a high expression of *PTP4A2* was associated with poor prognosis for both GBM and IDH-mutant gliomas (Fig. 2C; Supplementary Fig. S3C). All these results suggested a key role of *PTP4A2* in GBM development and aggressiveness, whereas *PTP4A1* expression was not a prognostic factor and *PTP4A3* expression was lower. To orientate the study on the potential functions of *PTP4A2* in GBM, we analyzed TCGA GBM data to find *PTP4A2*-correlated genes by performing enrichment in Gene Ontology terms. This correlation analysis identified several pathways potentially involved in *PTP4A2* function in patients with GBM (Fig. 2D). Among them, phagocytosis (GO:0006909) and leucocyte activation (GO:0050867) ranked the top biological processes, arguing for a role of *PTP4A2* in GBM progression through its putative role in the immune TME.

### PTP4A2 regulates tumor growth and apoptosis in a murine orthotopic GBM xenograft model

**Figure 3:**
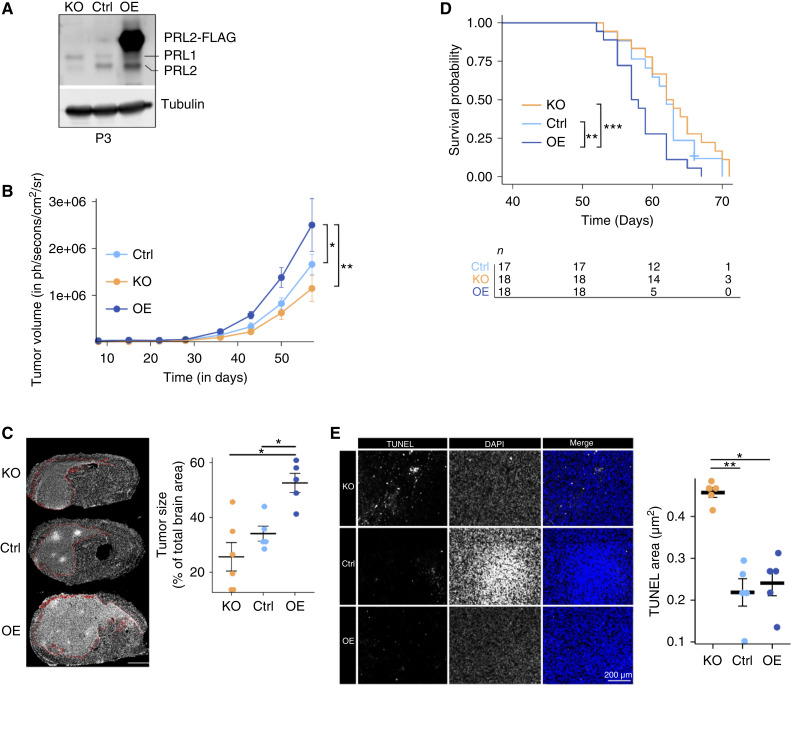
*In vivo* phenotype associated with *PTP4A2* modulation in P3 xenografts. **A,** Western blot analysis of Ctrl, *PTP4A2*-KO, and *PTP4A2*-OE P3 cells. **B,** Tumor volume assessed by bioluminescent imaging from orthotopically xenografted P3 spheroids (KO, Ctrl, and OE) over approximately 2 months after implantation. Kruskal–Wallis test followed by Dunn tests. *n* = 8 mice per group. **C,** Tumor size of P3 *PTP4A2*-KO, Ctrl, and *PTP4A2*-OE tumors. Mice were sacrificed at the same time point to compare the tumor area. *n* = 5 mice per group. **D,** Kaplan–Meier curves of survival (time to endpoint) of mice bearing KO, Ctrl, and OE tumors. Survival was not affected by the KO but was reduced by the OE. Log-rank test. The number of mice is indicated in the figure. **E,** TUNEL assay performed on sections from P3 Ctrl, *PRP4A2*-KO, and *PRP4A2*-OE tumors showing significantly more apoptosis in KO tumors compared with Ctrl and OE. Kruskal–Wallis test followed by Dunn tests. *n* = 5 mice per group.

We then investigated the phenotypical effects of the modulation of *PTP4A2* expression *in vivo*. We chose intracranial implantation of P3 cells which give rise to tumors that recapitulate the human histopathology and display interactions between tumor cells and their microenvironment (14). Unlike the 1123-Mes model, P3 orthotopic xenografts displayed invasive margins (Supplementary Fig. S4). We conducted orthotopic implantation of *PTP4A2*-KO, control (Ctrl), and *PTP4A2*-OE P3 spheroids in which tumor development was monitored for 2 to 3 months. The level of *PTP4A2* for each cell line is shown in Fig. 3A. Of note, the level of *PTP4A1* increased in *PTP4A2*-KO spheroids, indicating a compensatory mechanism as observed in other cell systems (22, 23). Tumor tissues were collected at the same time point for comparative studies, whereas each mouse was sacrificed at individual endpoints for survival experiments. Monitoring tumor growth by bioluminescence showed that *PTP4A2*-KO tumors tend to grow slower than Ctrl tumors (Fig. 3B). Conversely, *PTP4A2*-OE tumors developed more rapidly. This was also confirmed at the histologic level with the PTP4A2-OE tumor area covering more than 50% of the brain section area at its maximal size (Fig. 3C). Although mouse survival was not significantly affected by *PTP4A2*-KO, it was reduced in *PTP4A2*-OE tumor–bearing mice (Fig. 3D). As we suspected that the proliferation or apoptosis rate would explain the differences in tumor growth, we performed histologic staining of the proliferative marker KI67 in tumor tissues. This analysis showed that the modulation of *PTP4A2* expression did not affect tumor cell proliferation (Supplementary Fig. S5). We then performed a TUNEL assay and found that *PTP4A2*-KO tumors were more apoptotic than Ctrl and *PTP4A2*-OE tumors (Fig. 3E). Altogether, our results indicate that *PTP4A2* expression regulated GBM growth and apoptosis in a xenograft model.

### PTP4A2 deletion upregulates proinflammatory signals in the immune microenvironment of a GBM xenograft model

**Figure 4:**
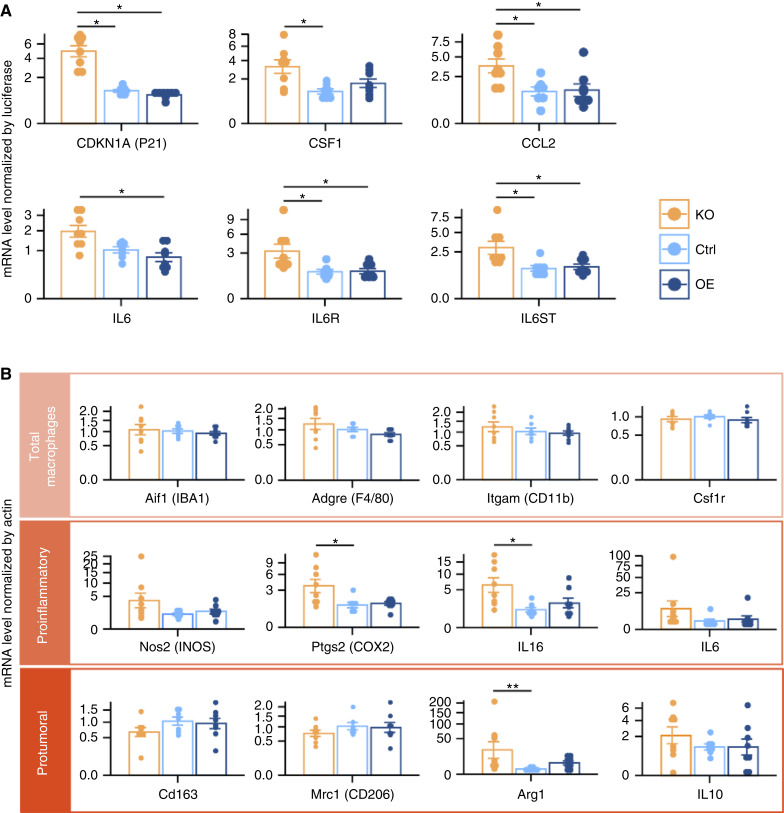
Transcriptional modulation of proinflammatory markers in P3 xenografts. **A,** Gene expression of different markers expressed by the human tumor cells inside the mouse brain, normalized by luciferase expression. qPCR primers were specific to human genes. **B,** Gene expression of different markers expressed by the TME, arranged by typical cell types and phenotypes: total macrophages, proinflammatory, and protumoral. Expression was normalized by actin expression. qPCR primers were specific to mouse genes. Kruskal–Wallis test followed by Dunn tests for all the figures. *n* = 7–8 mice per group

Based on the correlation analysis shown in Fig. 2D highlighting immune functions and the differences between the *in vitro* and *in vivo* phenotypes, we hypothesized that the TME plays a crucial role in *PTP4A2* oncogenic functions. We thus quantified gene expression and protein levels of different immune-related markers in *PTP4A2*-KO, Ctrl, and *PTP4A2*-OE tumors. Expression analysis by qPCR, using human-specific primers, showed a significant increase in the expression of some chemoattractant factors (i.e., CCL2 and CSF1) of the cell-cycle inhibitor P21 in the tumor cells of *PTP4A2*-KO xenografts (Fig. 4; Supplementary Fig. S6A and S6C). These changes were not observed when spheroids were cultured *in vitro* (Supplementary Fig. S7), supporting a prominent role of the TME in PTP4A2-dependent tumor growth. Expression analysis by qPCR, using mouse-specific primers, showed increased levels of proinflammatory cytokines and markers in the KO tumors (Fig. 4B; Supplementary Fig. S6B). These markers were associated with an antitumor M1-like phenotype in macrophages and microglia. Markers for total macrophages/microglia such as *Adgre* (F4/80) or *Aif1* (IBA1) were not differentially expressed, suggesting that *PTP4A2* expression did not affect macrophage accumulation inside the tumor. Protumor and immunosuppressor M2-like markers such as *Cd163* and *Mrc1* (CD206) were also not changed. Overall, at the transcriptional level, proinflammatory signals were increased in *PTP4A2*-KO tumors.

**Figure 5:**
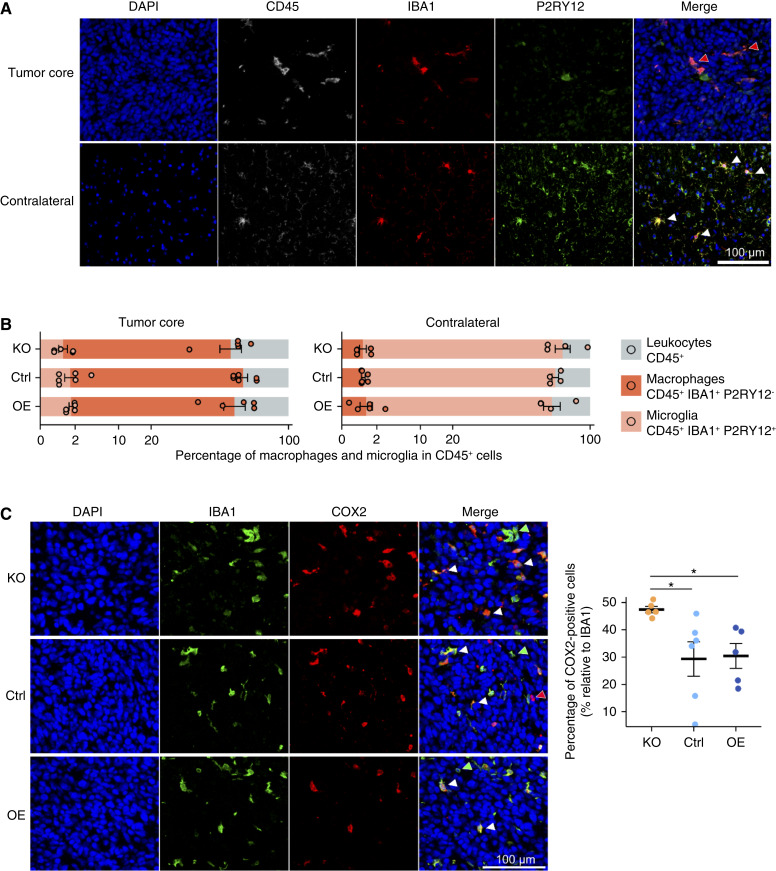
Phenotype of macrophages and microglia in P3 xenografts. **A,** Representative images of macrophages (CD45^+^ IBA1^+^ P2RY12^−^) and microglia (CD45^+^ IBA1^+^ P2RY12^+^) in the tumor core and in the contralateral brain of a control tumor. Red triangles indicate macrophages positive for both CD45 and IBA1 and negative for P2RY12. White triangles indicate microglia positive for CD45, IBA1, and P2RY12. **B,** Proportion of macrophages and microglia within the leukocyte population of the tumor core and contralateral brain of KO, Ctrl, and OE xenografts. **C,** Immunostaining of COX2 and IBA1 with representative images (left) and quantification (right). Kruskal–Wallis test followed by Dunn tests. White triangles indicate IBA1^+^ COX2^+^ cells, red triangles indicate IBA1^−^ COX2^+^ cells, and green triangles indicate IBA1^+^ COX2^−^ cells. *n* = 5–6 mice per group.

Next, we characterized myeloid-derived cells in *PTP4A2*-KO, Ctrl, and *PTP4A2*-OE xenografts by immunostaining. First, we assessed the proportion of macrophages, microglia, and neutrophils by triple immunofluorescence staining of IBA1, P2RY12, and Ly6G in P3 xenograft histologic sections. As expected, the main immune cell population was macrophages/microglia, positive for CD45 and IBA1, and accounting for 60% to 67% of immune cells (Fig. 5A and B). Neutrophils were less abundant and not statistically different in all tumors (Supplementary Fig. S8). In both *PTP4A2*-KO and -OE xenografts, the number of macrophages/microglia was similar to the controls (Fig. 5B). The tumor core was preferentially infiltrated by tumor-associated macrophages (TAM; i.e., IBA1^+^ P2RY12^−^) which accounted for 59% to 65% of CD45^+^ cells, whereas microglia represented less than 2%. In contrast, the parenchyma of the contralateral brain was preferentially infiltrated by microglia (Fig. 5B). The morphology of macrophages and microglia was amoeboid-like in the tumor core and more ramified in the contralateral brain (Fig. 5A). This is in line with previous reports showing that the ramified phenotype is typical for “resting” microglia and the amoeboid phenotype is seen in both microglia and macrophages and is associated with a more active state (24). We then assessed the macrophages/microglia ratio by histologic analysis for CD45^+^ IBA1^+^ P2RY12^−^ (macrophages) and CD45^+^ IBA1^+^ P2RY12^+^ (microglia) and found no differences between *PTP4A2*-KO, Ctrl, and *PTP4A2*-OE tumors. Importantly, when we examined the inflammatory status of macrophages/microglia in the tumors, we observed an increase in COX2-positive macrophages in KO tumors compared with control and OE tumors (Fig. 5C). These results and the qPCR data described above indicate that *PTP4A2*-KO tumors present an enhanced proinflammatory microenvironment.

### PTP4A2 deletion reduces tumor growth and polarizes macrophages toward a proinflammatory phenotype in a GBM syngeneic model

**Figure 6:**
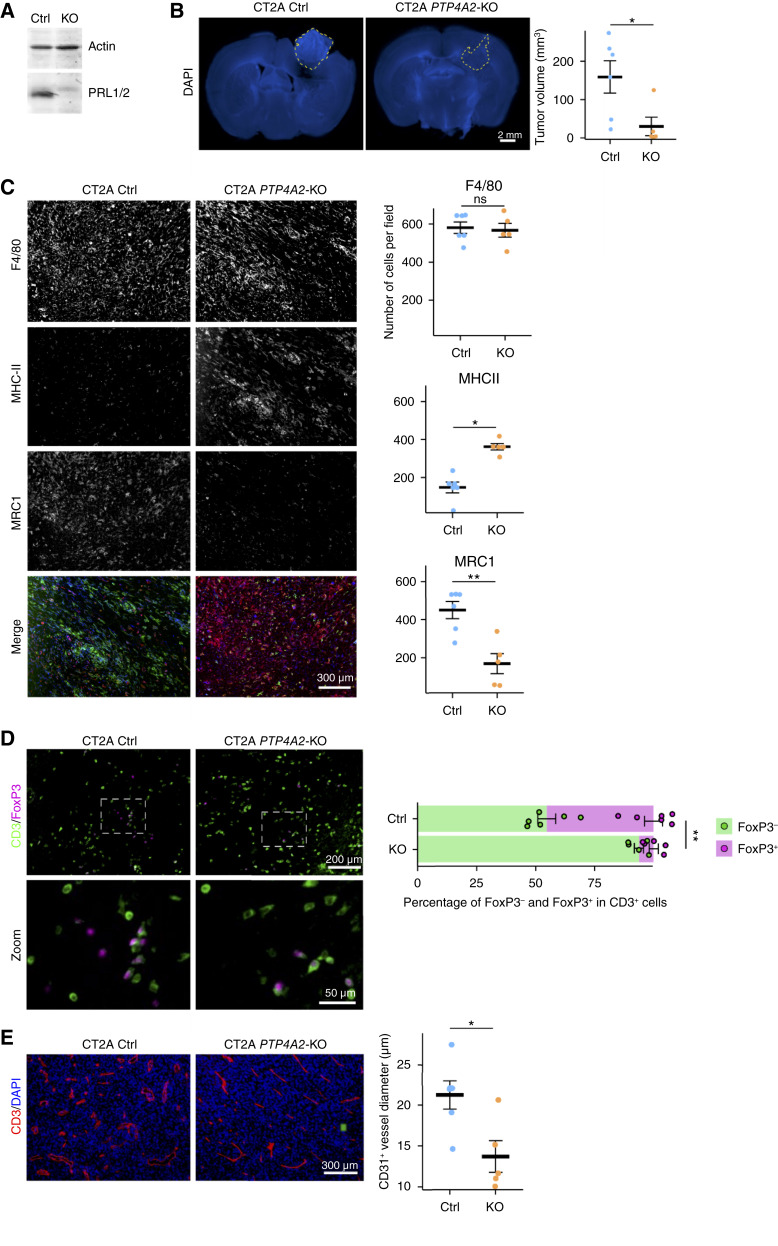
Phenotype of *PTP4A2*-KO and Ctrl tumors in the CT2A syngeneic model. **A,** Western blot analysis of Ctrl and *PTP4A2*-KO CT2A cells. **B,** Tumor volume with representative images of DAPI staining in CT2A tumor sections (left) and quantification (right). Permutation *t* test. **C,** Immunostaining of F4/80, MHC-II, and MRC1 with representative images (left) and quantification (right). **D,** Immunostaining of CD3 and FoxP3 with representative images (left) and quantification (right). Mann–Whitney U test. **E,** Immunostaining of CD31 and DAPI with representative images (left) and quantification (right). Permutation *t* test. *n* = 5–6 mice per group.

To further explore the effects of *PTP4A2* deletion on the tumor microenvironment, we used a syngeneic GBM mouse model. Engraftment of CT2A cells depleted for *PTP4A2* expression and their matching controls in C57Black6 immunocompetent mice showed delayed tumoral growth (Fig. 6A and B). Genetic depletion of *PTP4A2* induced a remodeling of the innate immune landscape, by upregulating MHC-II^+^ macrophages and downregulating MRC1^+^ macrophages, without altering the total number of F4/80-positive TAMs (Fig. 6C). The deletion of *PTP4A2* showed other immunosuppressive features with a decrease in CD3^+^ FoxP3^+^ regulatory T cells compared with control (Fig. 6D). Immunostaining of CD31 revealed a step toward normalization of the blood vessel network in PTP4A2-KO tumors highlighted by a less dysmorphic vasculature than that in control tumors (Fig. 6E). In line with the outcomes of the xenografts, *PTP4A2*-KO rewired the TME toward an antitumor phenotype.

### *In vitro* phenotypic analysis in *PTP4A2*-KO GBM cells leads to contrasting results

**Figure 7:**
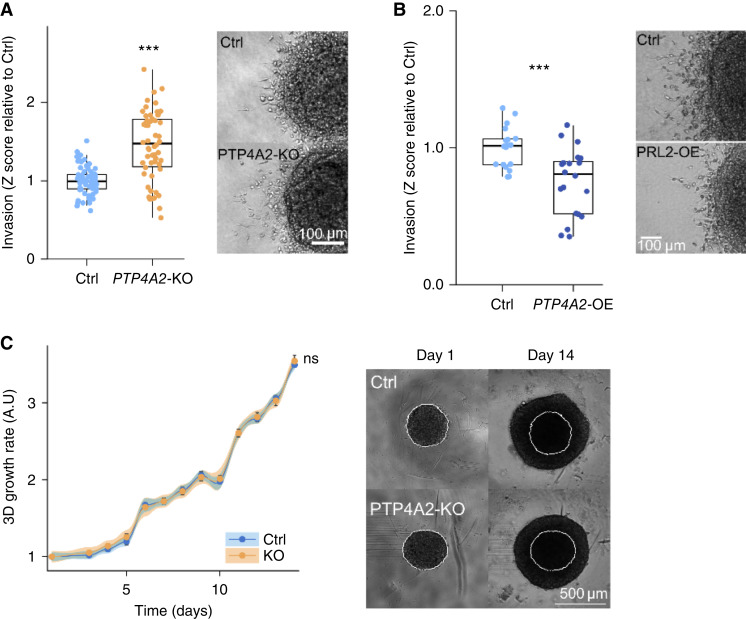
*In vitro* phenotype of P3 spheroids. **A,** Spheroid invasion assay in collagen I of Ctrl and *PTP4A2*-KO 1 and 2 P3 cells. The invasive area was measured after 24 hours. **B,** Spheroid invasion assay in collagen I of Ctrl and *PTP4A2*-OE. **C,** Proliferation assay of Ctrl and *PTP4A2*-KO P3 cells based on spheroid growth. Kruskal–Wallis test followed by Dunn tests.

To gain insights into *PTP4A2*-dependent mechanisms in GBM growth, we performed *in vitro* assays using human GBM spheroids. Control and *PTP4A2*-KO spheroids were included into collagen type I gel, and invasive areas were measured at 24 hours. P3 spheroid invasion was increased in the absence of *PTP4A2* and decreased when *PTP4A2* was overexpressed (Fig. 7A and B). *PTP4A2* depletion improved P3 cell adhesion on all tested coating (fibronectin, collagen type I, and Matrigel) as well as cell migratory capacities (Supplementary Fig. S9A and S9B). Surprisingly, knocking out *PTP4A2* in two other models, 1123-Mes and 157-PN, drastically decreased spheroid invasion (Supplementary Fig. S10A–S10D). P3 spheroid viability was measured by ATP content, and the growth rate was quantified by cell counting and spheroid size measurement. Both spheroid viability and growth were not affected by *PTP4A2* expression modulation (Fig. 7C; Supplementary Fig. S9C and S9D). This result contrasted with those obtained *in vivo*, suggesting that some microenvironment factors are essential for *PTP4A2* oncogenic function in GBM. Together, these *in vitro* results suggest that the effects of *PTP4A2* on tumor growth most likely involve the TME present in our *in vivo* xenograft model. TME dependence for *PTP4A2* function in other GBM cell lines (e.g., 1123-Mes and 157-PN) seems to be less, which may explain the contrasting findings. An additional explanation is that the effect on invasion is GBM subtype specific. In P3 cells, compaction, and not invasion, is favored when PRL2 is silenced.

## Discussion

The *PTP4A2* phosphatase has previously been linked to multiple cancer types (10), and we now report that its oncogenic functions also contribute to GBM progression. Using a xenograft model that recapitulates patient disease, we show that *PTP4A2* is involved in GBM growth under microenvironmental pressure. Deleting *PTP4A2* reduced tumor growth and increased apoptosis *in vivo*, and overexpressing *PTP4A2* promoted tumor growth. In contrast, its overexpression in P3 spheroids did not modulate proliferation and was inversely correlated with invasion *in vitro*. The dichotomy of *PTP4A2* functions between *in vitro* and *in vivo* revealed the crucial role of the TME in *PTP4A2* functions in GBM.

Although previous studies have already demonstrated the oncogenic role of *PTP4A2* in mouse models including breast cancer xenograft (11, 25) and T-cell leukemia (26), their results correlated with their *in vitro* observations. In our study, *PTP4A2* depletion or OE had mostly no consequence on *in vitro* cell proliferation/viability but a significant effect on tumor growth in the orthotopic GBM xenograft mouse model. To the best of our knowledge, this is the first report on the differential functions of *PTP4A2* in response to tumor microenvironmental pressure. However, Funato and colleagues (27) showed that *PTP4A3* expression enhanced the proliferation of noncancerous cells under acidic conditions, a parameter of microenvironmental pressure. If this mechanism applied to cancer cells, this could provide them with a survival advantage inside the TME.

A major finding of our study showed that *PTP4A2*-KO xenografts and/or syngeneic tumors displayed upregulation of proinflammatory markers including IL6, CSF1, and CCL2 at the transcriptional or protein level. As part of a cross-talk, the microenvironment of *PTP4A2*-KO tumors seemed more proinflammatory with the upregulation of immunosupportive macrophages. In line with the literature (28), macrophages were the most abundant myeloid cells in our xenograft model, which suggests that this proinflammatory signaling could be attributed to macrophages. TAMs are mainly immunosuppressive in GBM and participate in tumor progression (29–31). Therefore, TAMs are key players in the development of resistance to anticancer therapies (32). They are also plastic cells with the capacity to adopt different phenotypes in response to diverse stimuli (31, 33). Hence, the re-education of TAM toward a more proinflammatory and immunosupportive phenotype seems to be a promising therapeutic strategy, particularly in GBM (34). To know whether the shift of a macrophage phenotype is a cause or a consequence of the tumor apoptosis observed in our study, our data suggest that targeting *PTP4A2* could participate in the re-education of TAMs in immunosupportive macrophages. However, in our study, we did not infer a cause–consequence relationship between the observed apoptosis and increased proinflammatory signals, and this should be addressed in future studies.

Taking advantage of our immunocompetent GBM model, we found that not only the macrophages are differentially polarized in *PTP4A2*-KO tumors, but also T cells and blood vessels are affected. The deletion of *PTP4A2* decreased the proportion of CD3^+^ FoxP3^+^ regulatory T cells. Blood vessel caliber is decreased in *PTP4A2*-KO tumors. A similar normalization of tumor vessels was previously reported for other pathways responsible for TAM cytotoxic-to-immunosuppressive switch (30).

In addition to the key role of the TME in *PTP4A2* effects, heterogeneity most probably influences the regulation of *PTP4A2* differentially in distinct areas of the tumor. These areas are described as the perinecrotic core, the perivascular niche, and the invasive niche (35). We can expect that in each of them, *PTP4A2* expression is differentially modulated in response to nutrient availability, cellular neighborhood, and physical forces and thus plays various roles. As seen in the IVY-GAP data, *PTP4A2* is mainly upregulated in the perinecrotic zone and in pseudopalisading cells around.

*PTP4A2*-OE tumors had an accelerated growth compared with Ctrl tumors, and this phenotype was inversely correlated with mouse survival. However, these effects did not seem to involve apoptosis, proliferation, or TME adaptation, suggesting that other mechanisms contribute to the role of *PTP4A2* in GBM and remain to be uncovered. For example, the PRLs interact with the CNNM magnesium regulators (7, 36). The PRL/CNNM complex is linked to tumor progression through the accumulation of intracellular magnesium (26, 37, 38). Indeed, numerous magnesium-dependent enzymes play crucial roles in metabolic processes and signaling pathways that sustain cancer progression (39). Of note, Li and colleagues have recently reported that high *PTP4A2* expression downregulated the PTEN level by dephosphorylation, suggesting that *PTP4A2* supports oncogenic propensity of PTEN deletion in cancer (8). However, our main cellular model for this study, P3 cells, is PTEN deficient. This could explain the discrepancy observed between cell lines as 157-PN and 1123-Mes do not carry PTEN deletion (Supplementary Fig. S11).

To propose a translational application to our findings, we investigated the inhibition of all three PRLs in GBM cells. This allowed overcoming the potential functional redundancy among the PRL family members as we observed when we targeted only *PTP4A2*, which led to an increase in *PTP4A1* expression levels. We performed experiments using a specific PRL inhibitor that efficiently compromised GBM cell proliferation in a similar range previously reported (20). So far, the capacity of the JMS-053 compound to cross the blood–brain barrier has not been investigated yet. Nevertheless, our results suggest a potential therapeutic interest in PRL inhibition against GBM development. There are significant challenges associated with the inhibition of the PRL family including the substantial homology among PRLs, making it difficult to specifically target individual members of the family. Additionally, the conservation of the active site with other PTPs further complicates the development of effective inhibitors. Still, more data are emerging with regard to the potential use of PRL inhibitors in clinical settings. For example, PRL3-zumab, a first-in-class humanized antibody that specifically binds to PRL3 (40), demonstrated a good safety profile in the phase I trial (41) and has now progressed to phase II clinical trials. Whether a similar approach could be used for targeting PRL2 remains to be tested.

In conclusion, our work provides evidence for an involvement of PRL2 in GBM development (Supplementary Fig. S12). The effect of PRL2 on regulating GBM progression is dependent on the interaction with the TME. Although *PTP4A2* depletion did not significantly impact mouse survival in the xenograft model, it modulated the TME with an upregulation of proinflammatory signals and apoptosis. Nevertheless, increased levels of *PTP4A2* in tumor cells enhanced tumor growth and impaired mouse survival. In an immunocompetent host, *PTP4A2* depletion significantly impacted tumor growth and rewired the TME by affecting immunity and the vasculature.

### Novelty and limitations of the study

Our study shows that PRL2 is involved in GBM development and that it rewires the TME by affecting immunity and the vasculature. A limitation of the study is the lack of a detailed molecular mechanism to explain PRL2’s action in GBM cells. Potential mechanisms are, nevertheless, discussed. A second constraint is the specificity of pharmacologic inhibition for PRL family members, which to date is only achieved for PRL3. Thus, we were only able to use existing pharmacologic inhibitors that inhibit all three PRLs.

## Supplementary Material

Supplementary Table Supplemental Table

Supplementary Figure 1 Effect of pharmacological PRL inhibition

Supplementary Figure 2 Spheroid growth assay in the presence of pharmacological PRL inhibitors

Supplementary Figure 3 PTP4As expression in patients and cell lines

Supplementary Figure 4 Immunostainings of Cd45 and GFP labeling of leukocytes and tumor cells respectively

Supplementary Figure 5 K67 staining in knock-out and PRL2 overexpressing cells.

Supplementary Figure 6 Modulation of pro-inflammatory markers in P3 xenografts

Supplementary Figure 7 mRNA levels of different markers

Supplementary Figure 8 Proportion of neutrophils in CD45+ cells in P3 xenografts

Supplementary Figure 9 In vitro phenotype of P3 cells

Supplementary Figure 10 In vitro phenotype of 1123-Mes and 157-PN cells

Supplementary Figure 11 Western Blot analysis of cell lysates

Supplementary Figure 12 The figure depicts the diffuse glioma when PTP42A is deregulated. Left panel (PTP42A ko) and right panel (PTP42A overexpression, PTP42A-OE).
