# Supplementary material for: PTP4A2 Promotes Glioblastoma Progression and Macrophage Polarization under Microenvironmental Pressure
Source: Cancer Res Commun. 2024 Jul 11;4(7):1702–14. doi: 10.1158/2767-9764.CRC-23-0334 (PMC11238266; doi:10.1158/2767-9764.CRC-23-0334)
Supplement: Supplementary Table — Supplemental Table [file crc-23-0334_supplementary_table_suppst.pdf]

Supplementary Table 1: Antibodies used in this study

Target	Provider	Number
PRL2	Merck	05-1583
P2YRY2	Biolegend	Clone S16007D
Ki67	DAKO	M7240
Ly6G	BD Pharmigen	551459
CD45	R&D Systems	AF114
IBA1	Abcam	ab178846
COX2	Abcam	ab179800
FoxP3	Ebioscience/Invitrogen	14-5773-82
CD3	Proteintech	17617-1-ap
CD31	R&D Systems	AF3628
MRC1	R&D Systems	AF2535
F4/80	Santa Cruz	SC377009
MHCII	Ebioscience/Invitrogen	14-5321-82
