# Supplementary material for: PTP4A2 Promotes Glioblastoma Progression and Macrophage Polarization under Microenvironmental Pressure
Source: Cancer Res Commun. 2024 Jul 11;4(7):1702–14. doi: 10.1158/2767-9764.CRC-23-0334 (PMC11238266; doi:10.1158/2767-9764.CRC-23-0334)
Supplement: Supplementary Figure 1 — Effect of pharmacological PRL inhibition [file crc-23-0334_supplementary_figure_1_suppsf1.pdf]

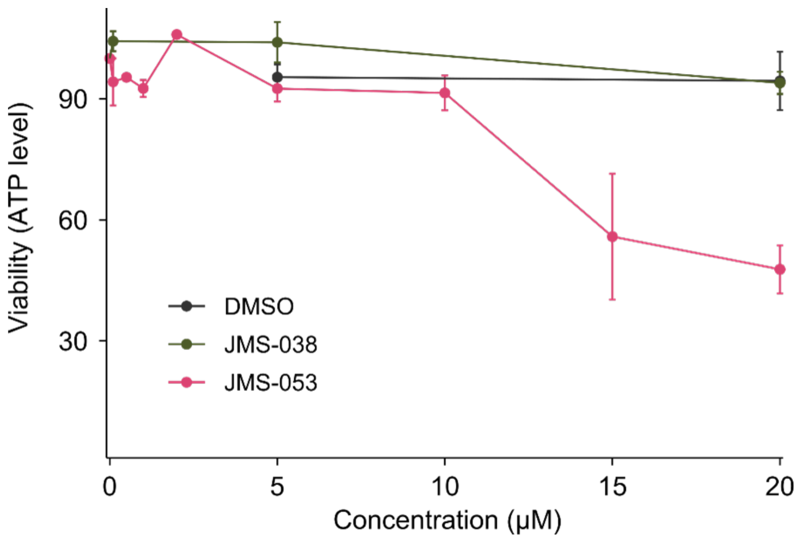

Supplementary Figure S1: **Effect of pharmacological PRL inhibition.**
Supplementary Figure S1 shows P3 cells viability after 5 days of treatment with JMS-053 (pink), JMS-038 (green) or DMSO (grey) as shown by ATP level. IC_{50} around 15 µM.
