# Supplementary material for: PTP4A2 Promotes Glioblastoma Progression and Macrophage Polarization under Microenvironmental Pressure
Source: Cancer Res Commun. 2024 Jul 11;4(7):1702–14. doi: 10.1158/2767-9764.CRC-23-0334 (PMC11238266; doi:10.1158/2767-9764.CRC-23-0334)
Supplement: Supplementary Figure 2 — Spheroid growth assay in the presence of pharmacological PRL inhibitors [file crc-23-0334_supplementary_figure_2_suppsf2.pdf]

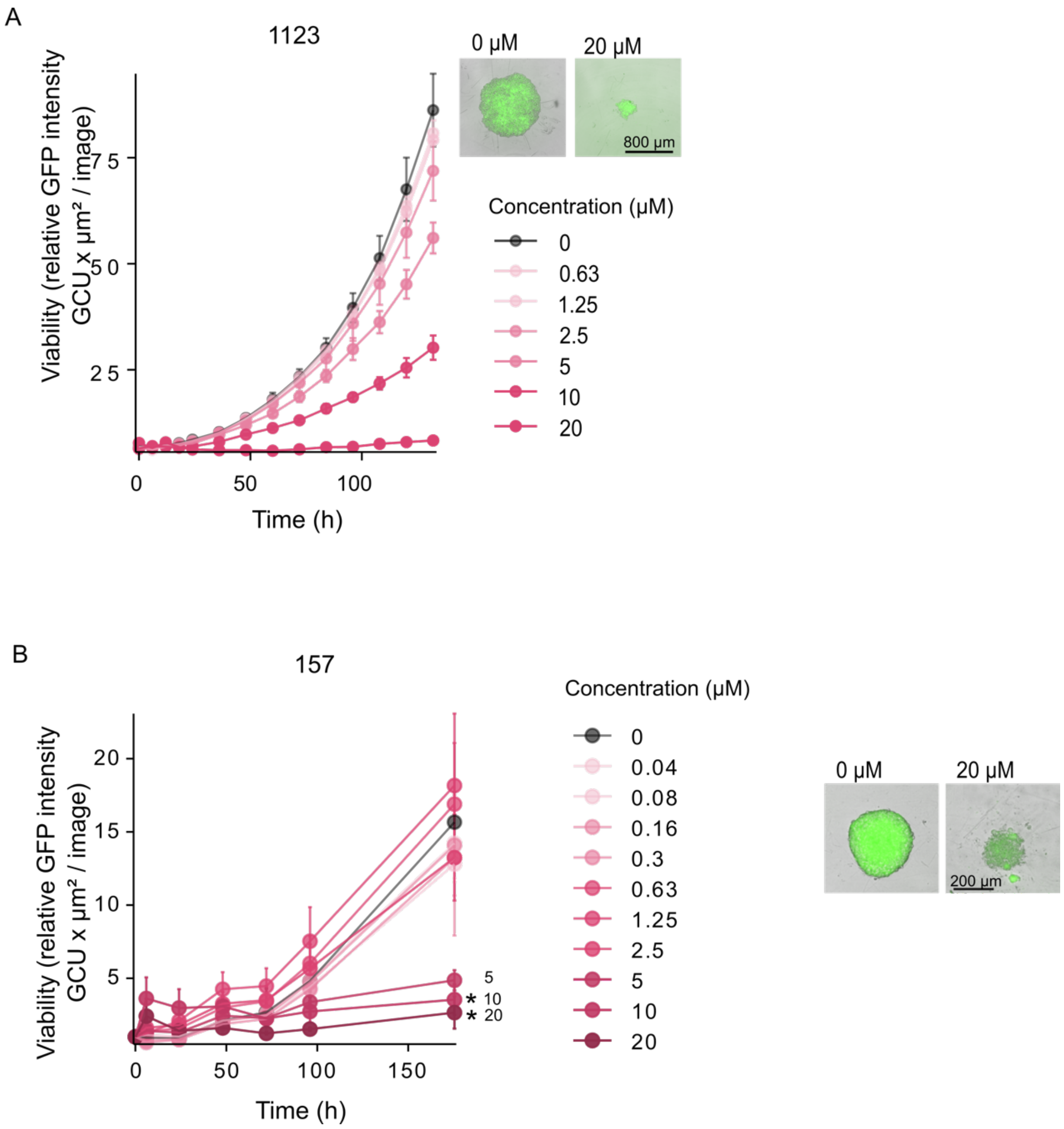

Supplementary Figure S2: **Spheroid growth assay in the presence of pharmacological PRL inhibitors.** Inhibition of the three PRLs by JMS-053 in 1123-Mes and 157-PN spheroids. **A**) Spheroid growth over 6 days of 1123-Mes cells treated with the increasing doses of PRL inhibitor JMS-053 (pink) or the vehicle DMSO (grey). n = 2. **B**) Spheroid growth over 7 days of 157-PN cells treated with increasing doses of PRL inhibitor JMS-053 (pink) or the vehicle DMSO (grey). n = 3. Kruskal-Wallis test followed by Dunn test.
