# Supplementary material for: PTP4A2 Promotes Glioblastoma Progression and Macrophage Polarization under Microenvironmental Pressure
Source: Cancer Res Commun. 2024 Jul 11;4(7):1702–14. doi: 10.1158/2767-9764.CRC-23-0334 (PMC11238266; doi:10.1158/2767-9764.CRC-23-0334)
Supplement: Supplementary Figure 3 — PTP4As expression in patients and cell lines [file crc-23-0334_supplementary_figure_3_suppsf3.pdf]

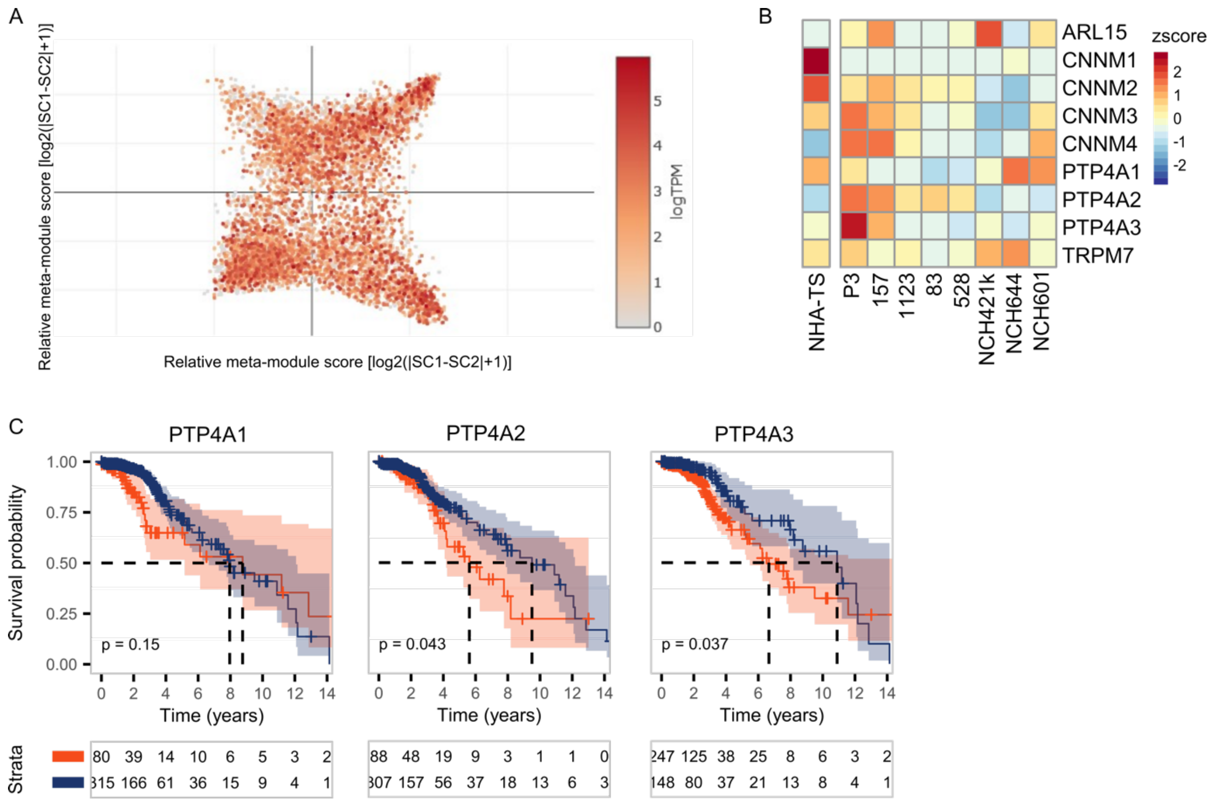

Supplementary Figure S3: **PTP4As expression in patients and cell lines**. **A**) PTP4A2 expression in cellular state hierarchy plot. Two-dimensional representation of cellular states. Quadrants: AC-like (bottom left), MES-like (bottom right), OPC-like (top left), NPC-like (top right). scRNAseq data from Neftel et al (13). **B**) mRNA expression of *PTP4A2* and associated genes in different GB cells compared to immortalized normal human astrocytes NHA-TS quantified by qPCR. In this study we used mainly P3 cells, 157-PN and 1123-Mes cells. All 3 cell lines have an increased expression of *PTP4A2*. **C**) Kaplan-Meier curve of overall survival of IDH-mutant gliomas of patients were split according to the expression level of *PTP4A1*, *2* and *3* (from TCGA Low Grade Gliomas).
