# Supplementary material for: PTP4A2 Promotes Glioblastoma Progression and Macrophage Polarization under Microenvironmental Pressure
Source: Cancer Res Commun. 2024 Jul 11;4(7):1702–14. doi: 10.1158/2767-9764.CRC-23-0334 (PMC11238266; doi:10.1158/2767-9764.CRC-23-0334)
Supplement: Supplementary Figure 4 — Immunostainings of Cd45 and GFP labeling of leukocytes and tumor cells respectively [file crc-23-0334_supplementary_figure_4_suppsf4.pdf]

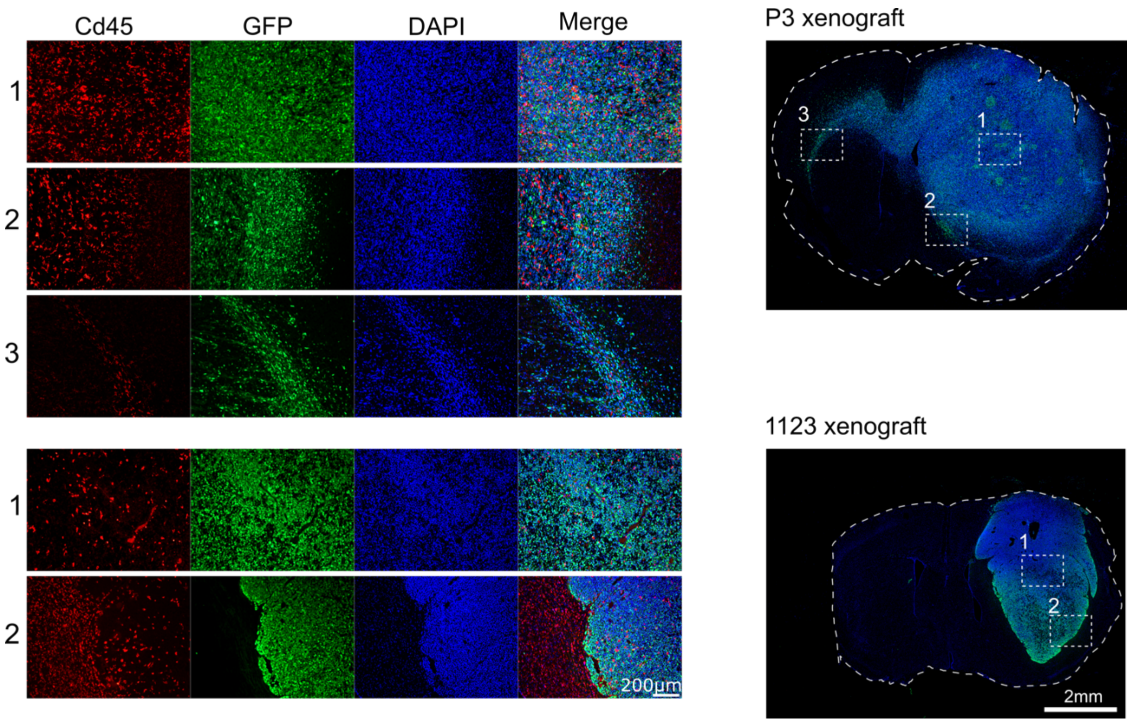

Supplementary Figure S4: **Immunostainings of Cd45 and GFP labeling of leukocytes and tumor cells respectively.** P3 xenograft (top) and 1123-Mes xenograft (bottom). Zooms on the borders and center of the tumors are displayed on the left and numbered accordingly.
