# Supplementary material for: PTP4A2 Promotes Glioblastoma Progression and Macrophage Polarization under Microenvironmental Pressure
Source: Cancer Res Commun. 2024 Jul 11;4(7):1702–14. doi: 10.1158/2767-9764.CRC-23-0334 (PMC11238266; doi:10.1158/2767-9764.CRC-23-0334)
Supplement: Supplementary Figure 5 — K67 staining in knock-out and PRL2 overexpressing cells. [file crc-23-0334_supplementary_figure_5_suppsf5.pdf]

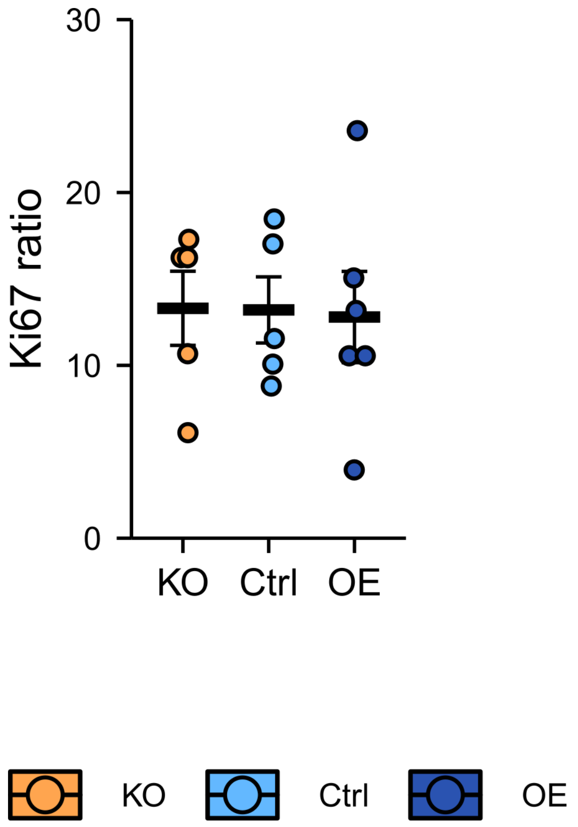

Supplementary Figure S5: **K67 staining in knock-out and PRL2 overexpressing cells.** Proportion of KI67 positive area relative to nucleus area of P3 xenografts (KI67 on DAPI expressed in %). Kruskal test, p-value ns.
