# Supplementary material for: PTP4A2 Promotes Glioblastoma Progression and Macrophage Polarization under Microenvironmental Pressure
Source: Cancer Res Commun. 2024 Jul 11;4(7):1702–14. doi: 10.1158/2767-9764.CRC-23-0334 (PMC11238266; doi:10.1158/2767-9764.CRC-23-0334)
Supplement: Supplementary Figure 6 — Modulation of pro-inflammatory markers in P3 xenografts [file crc-23-0334_supplementary_figure_6_suppsf6.pdf]

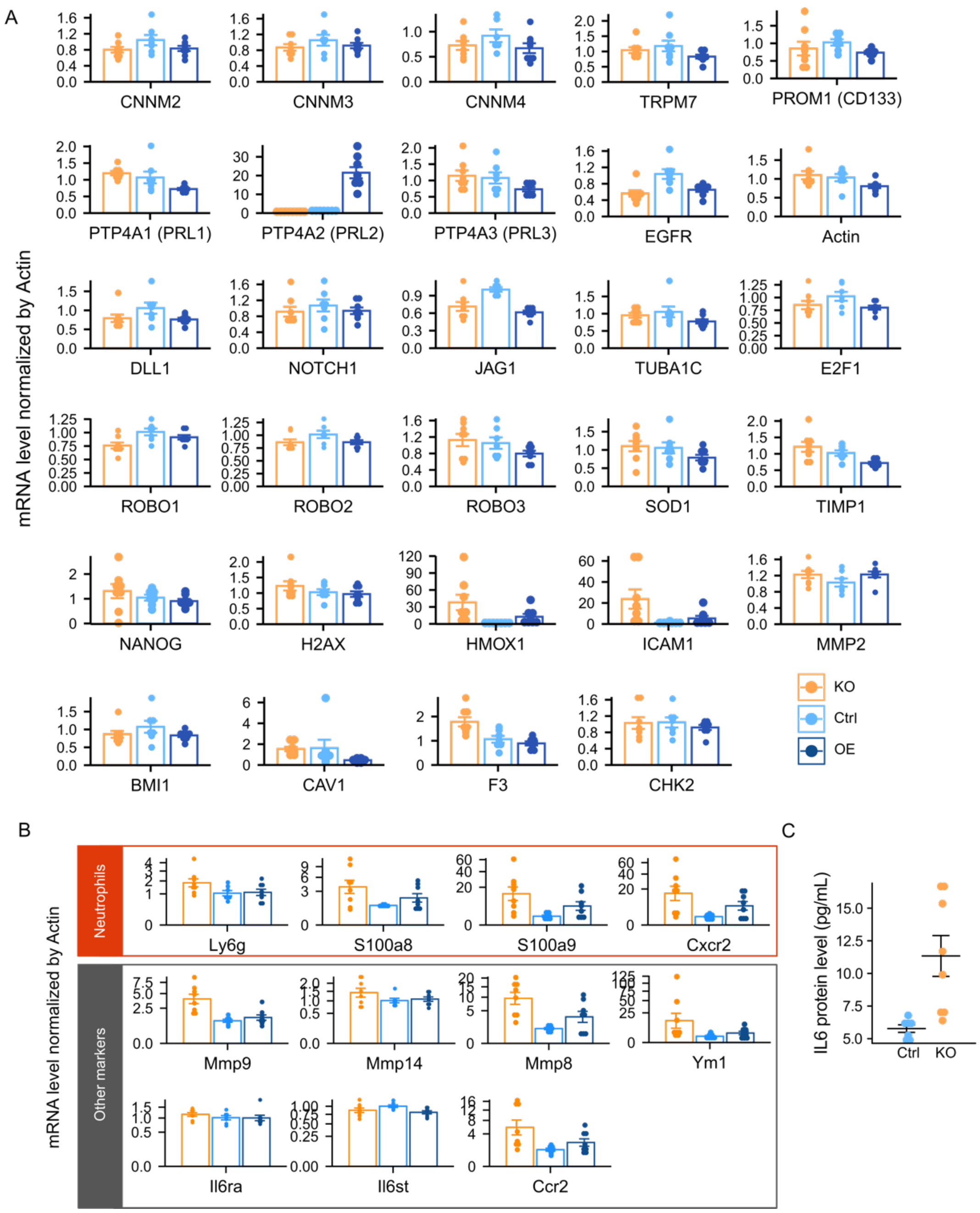

Supplementary Figure S6: **Modulation of pro-inflammatory markers in P3 xenografts**. **A**) Gene expression of different markers expressed by the human tumor cells inside the mouse brain, normalized by Luciferase expression. **B**) Gene expression of different markers expressed by the TME. **C**) IL-6 levels in Ctrl and KO tumor extracts quantified by ELISA, confirming the qPCR result.
