# Supplementary material for: PTP4A2 Promotes Glioblastoma Progression and Macrophage Polarization under Microenvironmental Pressure
Source: Cancer Res Commun. 2024 Jul 11;4(7):1702–14. doi: 10.1158/2767-9764.CRC-23-0334 (PMC11238266; doi:10.1158/2767-9764.CRC-23-0334)
Supplement: Supplementary Figure 7 — mRNA levels of different markers [file crc-23-0334_supplementary_figure_7_suppsf7.pdf]

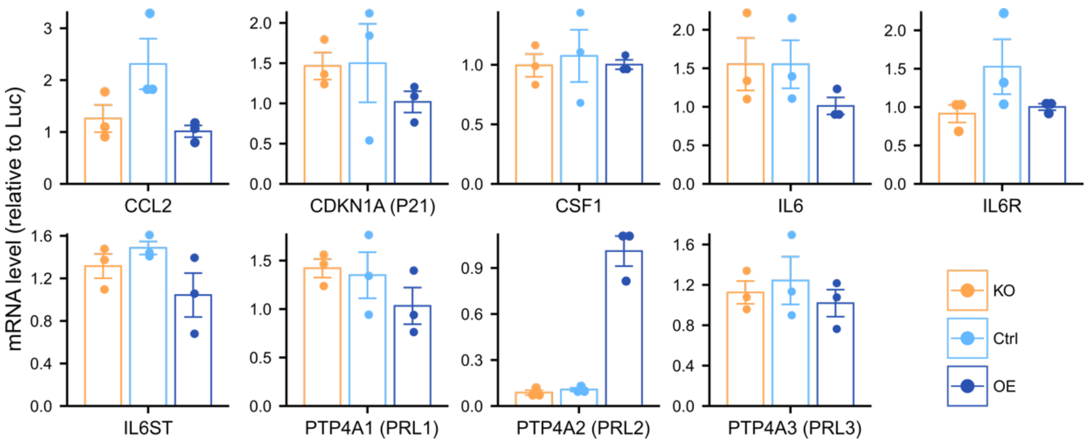

Supplementary Figure S7: **mRNA levels of different markers**. mRNA levels of different markers expressed by the P3 spheroids *in vitro* quantified by qPCR.
