# Supplementary material for: PTP4A2 Promotes Glioblastoma Progression and Macrophage Polarization under Microenvironmental Pressure
Source: Cancer Res Commun. 2024 Jul 11;4(7):1702–14. doi: 10.1158/2767-9764.CRC-23-0334 (PMC11238266; doi:10.1158/2767-9764.CRC-23-0334)
Supplement: Supplementary Figure 8 — Proportion of neutrophils in CD45+ cells in P3 xenografts [file crc-23-0334_supplementary_figure_8_suppsf8.pdf]

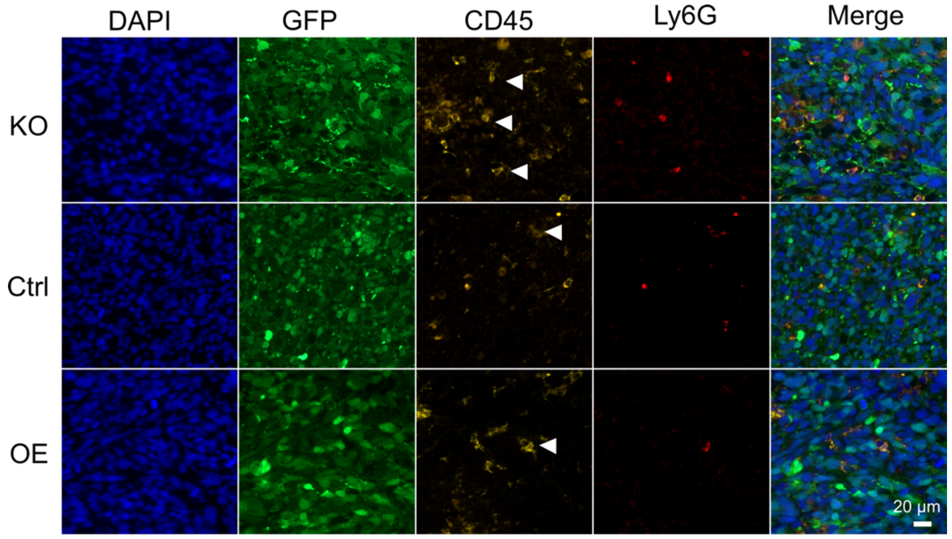

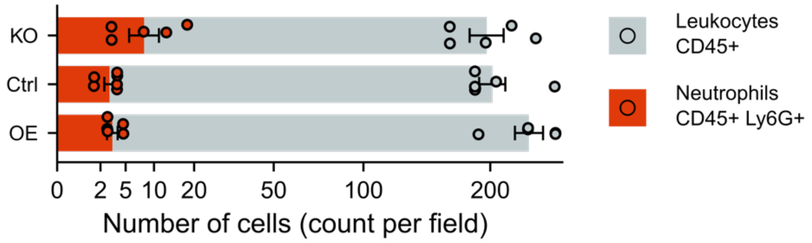

Supplementary Figure S8: **Proportion of neutrophils in CD45+ cells in P3 xenografts.** Representative images (top) and quantification (bottom). White triangles indicate neutrophils (CD45+ Ly6G+).
