# Supplementary material for: PTP4A2 Promotes Glioblastoma Progression and Macrophage Polarization under Microenvironmental Pressure
Source: Cancer Res Commun. 2024 Jul 11;4(7):1702–14. doi: 10.1158/2767-9764.CRC-23-0334 (PMC11238266; doi:10.1158/2767-9764.CRC-23-0334)
Supplement: Supplementary Figure 9 — In vitro phenotype of P3 cells [file crc-23-0334_supplementary_figure_9_suppsf9.pdf]

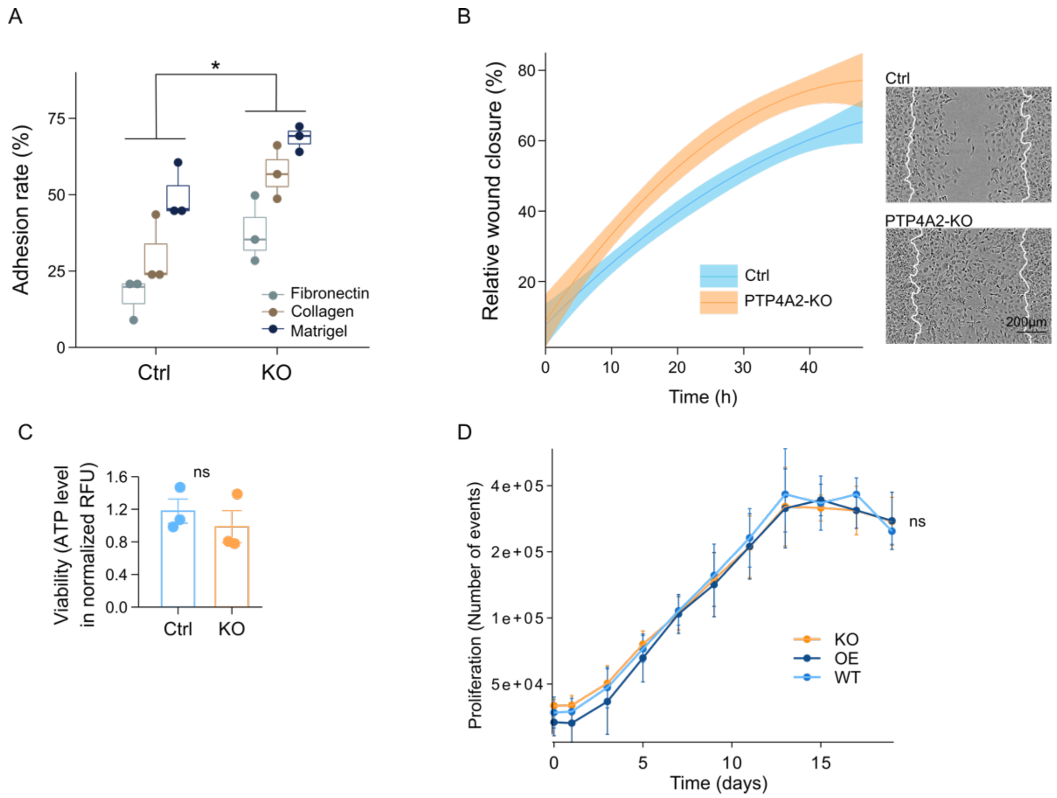

Supplementary Figure S9: ***In vitro* phenotype of P3 cells. A**) Adhesion assay of Ctrl and KO P3 cells. Cells were seeded on matrix coating (fibronectin, collagen and Matrigel) and washed after 20 min. Cells attached to the plate were lysed and quantified by colorimetric assay and adhesion. Adhesion rate is calculated by the ratio of adhered cells at 20 min / total cells at 4h then evaluated with Kruskal-Wallis test followed by Dunn tests. **B**) 2D migration measured in wound healing assay using Incucyte. **C**) Viability of Ctrl and KO P3 cells measured by ATP level. Mann–Whitney U test. **D**) Proliferation assay of Ctrl, KO, and OE P3 cells based on cell counting by flow cytometry then evaluated with Kruskal-Wallis test followed by Dunn tests. n = 3 experiments per panel.
