# Supplementary material for: PTP4A2 Promotes Glioblastoma Progression and Macrophage Polarization under Microenvironmental Pressure
Source: Cancer Res Commun. 2024 Jul 11;4(7):1702–14. doi: 10.1158/2767-9764.CRC-23-0334 (PMC11238266; doi:10.1158/2767-9764.CRC-23-0334)
Supplement: Supplementary Figure 10 — In vitro phenotype of 1123-Mes and 157-PN cells [file crc-23-0334_supplementary_figure_10_suppsf10.pdf]

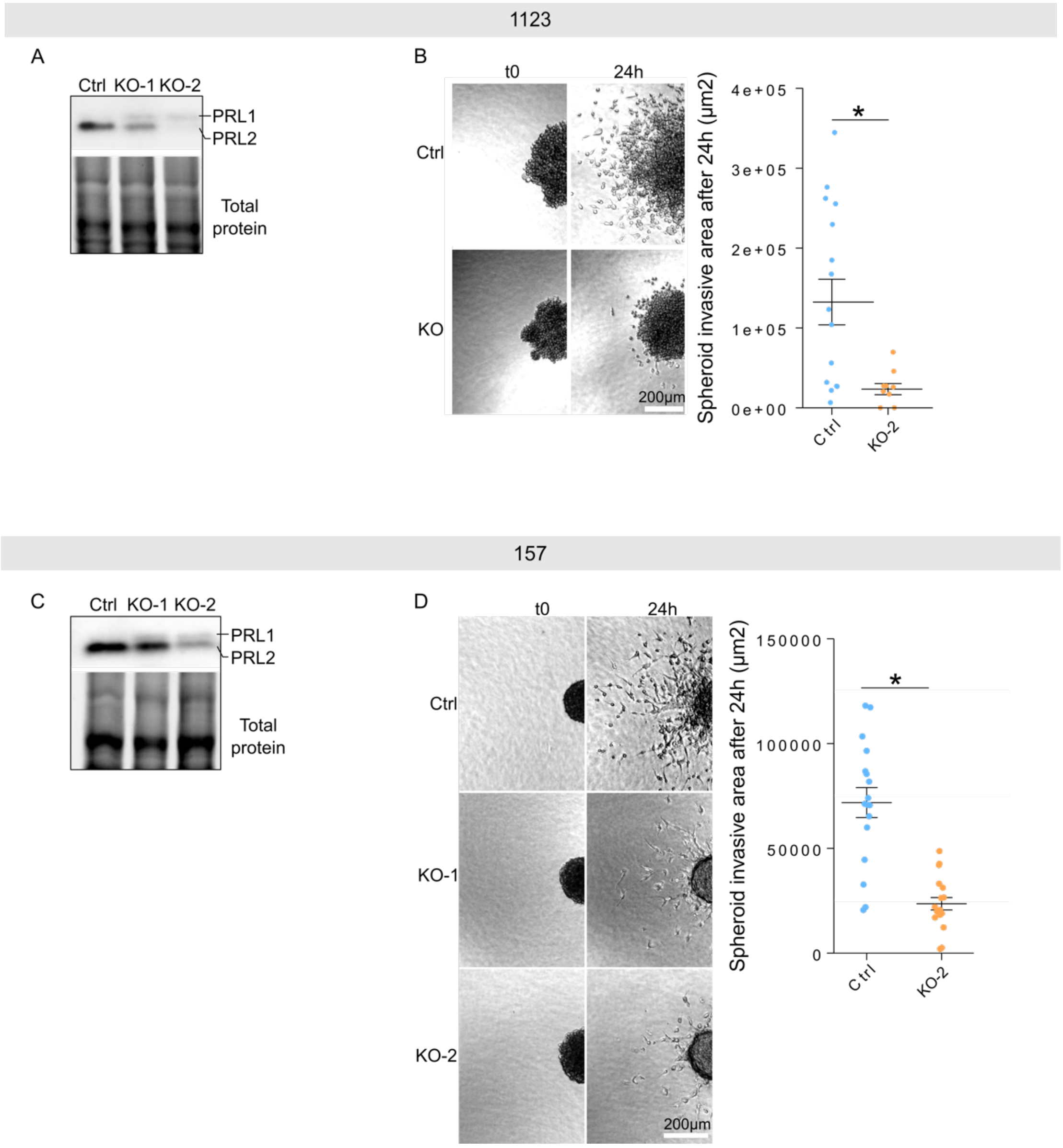

Supplementary Figure S10: ***In vitro* phenotype of 1123-Mes and 157-PN cells**. **A**) WB analysis in Ctrl, *PTP4A2*-KO-1, and KO-2 of 1123-Mes cells. **B**) Spheroid invasion assay in collagen I of Ctrl (n = 14) and *PTP4A2*-KO-2 (n = 9) 1123-Mes. **C**) WB analysis Ctrl, *PTP4A2*-KO-1, and KO-2 of 157-PN cells. **D**) Spheroid invasion assay in collagen I of Ctrl (n = 16) and *PTP4A2*-KO-2 (n = 16) 157-PN. Mann–Whitney U test was used for statistical evaluation.
