# Supplementary material for: PTP4A2 Promotes Glioblastoma Progression and Macrophage Polarization under Microenvironmental Pressure
Source: Cancer Res Commun. 2024 Jul 11;4(7):1702–14. doi: 10.1158/2767-9764.CRC-23-0334 (PMC11238266; doi:10.1158/2767-9764.CRC-23-0334)
Supplement: Supplementary Figure 11 — Western Blot analysis of cell lysates [file crc-23-0334_supplementary_figure_11_suppsf11.pdf]

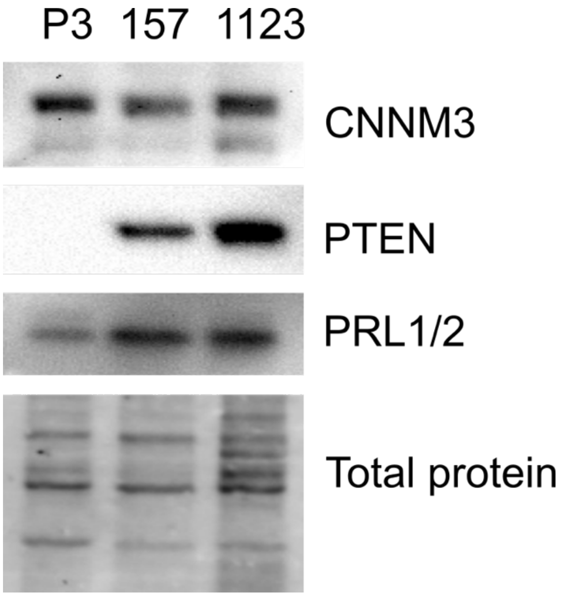

Supplementary Figure S11: **Western Blot analysis of cell lysates**. Supplementary Figure S11 shows the Western blot analysis of cell lysates from P3, 157-PN and 1123-Mes cells showing protein levels of CNNM3, PTEN and PRL1 and 2.
