# Supplementary material for: PTP4A2 Promotes Glioblastoma Progression and Macrophage Polarization under Microenvironmental Pressure
Source: Cancer Res Commun. 2024 Jul 11;4(7):1702–14. doi: 10.1158/2767-9764.CRC-23-0334 (PMC11238266; doi:10.1158/2767-9764.CRC-23-0334)
Supplement: Supplementary Figure 12 — The figure depicts the diffuse glioma when PTP42A is deregulated. Left panel (PTP42A ko) and right panel (PTP42A overexpression, PTP42A-OE). [file crc-23-0334_supplementary_figure_12_suppsf12.pdf]

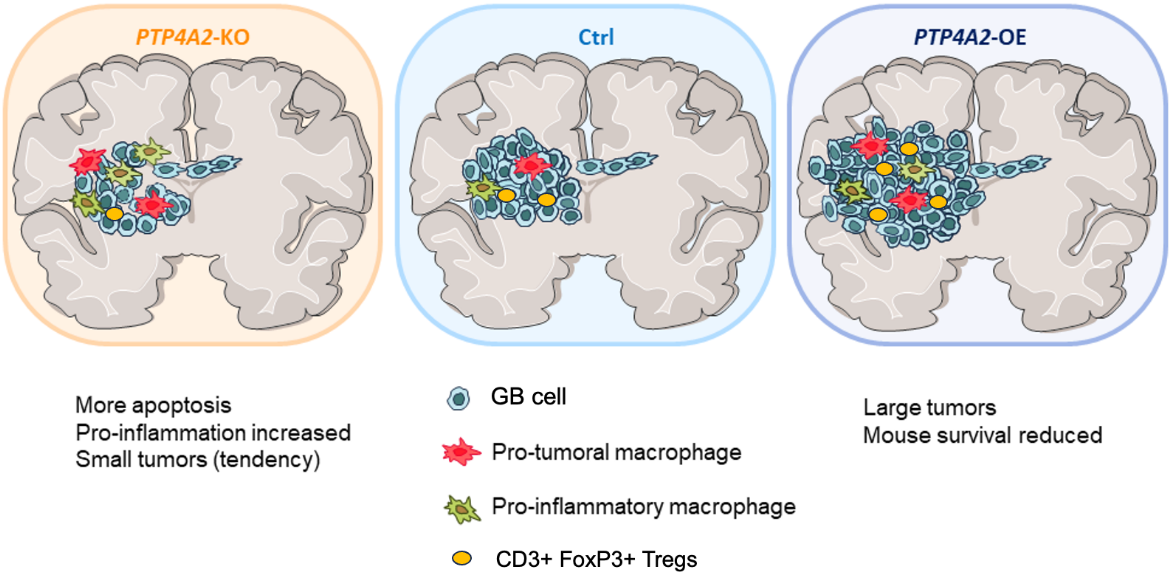

Supplementary Figure 12 : Graphical Abstract. The figure depicts the diffuse glioma when PTP42A is deregulated. Left panel (PTP42A ko) and right panel (PTP42A overexpression, PTP42A-OE).
